# Influence of Rust Layer on Corrosion-Critical Humidity in Outdoor Environments Based on Corrosion Sensors

**DOI:** 10.3390/ma18102299

**Published:** 2025-05-15

**Authors:** Qing Li, Xinyu Wang, Zibo Pei, Kui Xiao, Xiaojia Yang, Xuequn Cheng

**Affiliations:** Institute of Advanced Materials and Technology, University of Science and Technology Beijing, Beijing 100083, China; 18030340508@163.com (Q.L.); 13177190667@163.com (X.W.); peizibo1992@163.com (Z.P.); xiaokui@ustb.edu.cn (K.X.)

**Keywords:** corrosion monitoring, atmospheric corrosion, critical humidity, weathering steel

## Abstract

In this study, the Fe/Cu-based two-electrode corrosion monitoring technique was employed to monitor the long-term atmospheric corrosion of carbon steel at five different outdoor sites within the China National Environmental Corrosion Platform. Based on the fitted monitoring data, the variation trend of corrosion-critical humidity as a function of exposure time at different monitoring locations was obtained. The cross-sectional rust layer of corrosion coupons from different experimental periods at each location was characterized using scanning electron microscopy and Raman spectroscopy to identify variations in the thickness and phase composition of the carbon steel rust layer. The influence of rust layer thickness and phase structure on the critical humidity of carbon steel in atmospheric environments was investigated. Finally, the corrosion resistance of weathering steel in Tianjin, China, was validated using corrosion monitoring techniques, and the corrosion mechanism of weathering steel was elucidated by analyzing the influence of the acquired rust layer phase structure on the critical humidity of carbon steel in atmospheric environments.

## 1. Introduction

Corrosion, as a destructive but easily overlooked problem, has become one of the important factors affecting the sustainable development of the national economy and society [1]. Among them, atmospheric corrosion in the natural environment is the most common form of corrosion. In the study of outdoor atmospheric environments, the concept of corrosion-critical humidity has long been used to refer to the relative humidity (RH) in the air when atmospheric corrosion occurs [2,3]. Van den Steen et al. [4] added magnesium chloride to the metal surface, and the surface appeared wet at 34% RH, while NaCl requires 77% RH to produce the same effect. This critical RH that makes the metal surface become wet is named critical humidity. Mehrdad [5] refers to the RH corresponding to the corrosion current detection value of 50 nA as critical humidity. It can be seen that the critical humidity of atmospheric corrosion is not the RH when corrosion occurs but the RH value in the atmosphere when atmospheric corrosion reaches a certain degree. Steel materials are among the primary materials used in industrial production and construction, while their relatively poor corrosion resistance has always been a focal issue in the field of corrosion science. However, traditional corrosion detection methods, such as polarization curves, require an artificial solution environment for measurement, making it difficult to obtain the critical humidity of low-alloy steel in outdoor atmospheric environments in situ. Due to the lack of outdoor corrosion monitoring methods, it remains challenging to investigate the influence of rust layers on wetting time in outdoor environments. Therefore, ISO 9223 [6] roughly defines the time when metal undergoes thin liquid film corrosion as the duration when the temperature is >0 °C and RH > 80%, considering only the impact of environmental factors on wetting time [7].

The atmospheric corrosion monitor (ACM) can reflect the real-time changes in the corrosiveness of the environment where the sensor is located by monitoring the corrosion current or impedance information on the surface of the sensor under the thin liquid film of the atmosphere and can also compare the corrosion resistance of different low-alloy steels by changing the anode metal material of the sensor [8]. Commonly used corrosion monitoring technologies include resistance probe [9,10], AC impedance [11,12], quartz crystal microbalance [13], double-electrode corrosion couple [14,15] and others. Among these, the resistance probe technique is not limited by the corrosive environment and medium where the material is located and is suitable for long-term corrosion monitoring. However, some studies have shown that the measured resistance value in a low corrosion environment was 4.89 times the actual value [9], indicating weak monitoring sensitivity. Based on the principle of electrochemical impedance spectroscopy, the AC impedance technique can reflect many useful electrochemical information about thin liquid film and materials in the corrosion process. However, it is limited by certain requirements for the stability of the reaction system during AC impedance measurement, so that the dynamics of the atmospheric environment make the measurement results slightly different from the actual situation. It is not applicable to situations where the RH in the environment is less than 30% [12]. Although quartz crystal microbalances can monitor corrosion at the nm level, they have extremely high requirements for monitoring materials and can only be used for short-term monitoring [13]. The two-electrode corrosion monitoring technique combines the advantages of non-destructive monitoring, high sensitivity, and long-term monitoring of more than one year and can reflect the real-time corrosion status of the material in a dynamic outdoor environment [14], which provides the possibility for describing the critical humidity of material corrosion.

Pei et al. [16] found from the perspective of data that, compared with various environmental factors, the growth of the rust layer has an increasingly significant impact on the atmospheric corrosion of carbon steel in the outdoor environment and gradually occupies a dominant role. In general, the rust layer of carbon steel is mainly composed of protective α-FeOOH and active γ-FeOOH and can promote the corrosion of low-alloy steel in the early stage and inhibit it in the later stage in the solution environment [17], with the overall trend of inhibiting corrosion on the macro time scale in years [18,19]. Through corrosion monitoring data, Li et al. [20] found that outdoor dynamic atmospheric corrosion can be divided into two states: rainfall/non-rainfall. The promotion/inhibition effect of the rust layer on atmospheric corrosion in non-rainfall conditions is still unclear. Outdoor atmospheric corrosion of carbon steel produces a complex array of oxides and hydroxides, including Fe_3_O_4_, α-FeOOH, γ-FeOOH, β-FeOOH, and Fe_5_HO_8_·4H_2_O, alongside amorphous phases, with environmental factors like chloride ions driving compositional shifts—α-FeOOH enhances protective stability, while β-FeOOH accelerates degradation due to its electrochemical reactivity [21].

In order to explore the influence of the rust layer on the critical humidity of corrosion of structural steel, the Fe/Cu type two-electrode corrosion monitoring technique was used to monitor the atmospheric corrosion of carbon steel for a long time at five monitoring sites in different outdoor environments of the national environmental corrosion platform in China (Tianjin, Dunhuang, Jiangjin, Lhasa, and Qionghai) [20]. Within one year, about 2.5 million corrosion monitoring data and quality loss data were collected; based on the real-time corrosion current data monitored, the trend of corrosion-critical humidity with exposure time in five locations was obtained. According to the cross-section of the rust layer of the corrosion coupons for different experimental cycles in each location, the difference in the thickness and phase composition of the carbon steel rust layer in different locations was characterized by scanning electron microscopy and Raman spectroscopy, and the effects of the rust layer thickness and phase structure on the critical humidity of the carbon steel atmospheric environment were explored by combining the above test results. Finally, through the corrosion monitoring technique, the corrosion resistance of weathering steel was verified, and the corrosion resistance mechanism of weathering steel was explained through the influence of the phase structure of the rust layer on the critical humidity of the carbon steel atmospheric environment.

## 2. Materials and Methods

### 2.1. Materials

In this test, Q235 carbon steel and Q420 weathering steel from five sites—Tianjin, Dunhuang, Jiangjin, Lhasa, and Qionghai—were used as research subjects. These steels are commonly used in bridge construction and were manufactured by Nanjing Iron and Steel Group, following the standard GB/T 41756-2022 [22]. Their chemical compositions are shown in Table 1.

### 2.2. Corrosion Monitoring Technology

**Figure 1:**
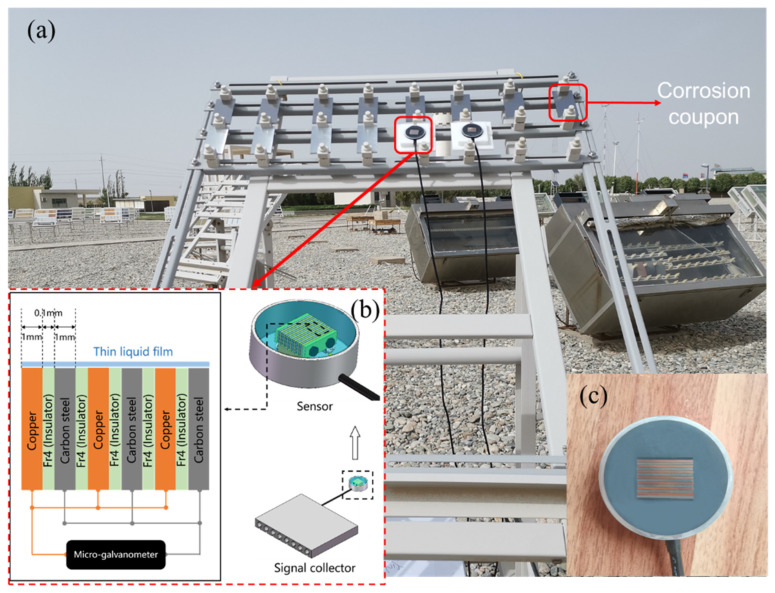
Installation site (**a**) of corrosion monitor sensor and corrosion coupons at Dunhuang experimental station (**b**) schematic diagram; (**c**) physical diagram [20].

The schematic diagram (Figure 1b) and physical diagram (Figure 1c) of the corrosion sensor in this experiment are shown in Figure 1. At each location, corrosion sensors and five parallel Q235 steel specimens and Q420 steel specimens (100 mm × 50 mm × 5 mm) are mounted more than 1 m above the ground and tilted 45° to the south. Temperature and relative humidity sensors are placed next to the corrosion sensor and exposed to the same corrosive environment. The sensor consists of 7 metal electrode sheets, with the anode metal being low-alloy steel and the cathode metal being pure copper (mass purity > 99.7%), and screw hole positions are reserved in the center of the structure. Temperature and relative humidity sensors are placed next to the corrosion sensor and exposed to the same corrosive environment. After the assembly is completed, the structure is fixed by insulating screws, and the metal electrode and the insulating plate are tightly compacted together to ensure that the spacing of the galvanic couple pair is accurately controlled by the insulating plate with a thickness of 0.1 mm. Then, the wire is led out from the back end of the cathode metal and the anode metal by crimping through the non-insulating screw to form a loop to ensure that the contact point between the electrode and the wire is minimal. Subsequently, the structure is potted with epoxy glue, and the surface is polished with 1200# silicon carbide sandpaper to complete the preparation of corrosion sensors. Finally, the corrosion sensor parameters of this test are as follows: anode area 21 mm × 1 mm × 7 mm, cathode to anode area ratio 1:1, insulation sheet using epoxy glass fiber board (grade FR4), and thickness of 0.1 mm [16].

### 2.3. Exposure Experiments

**Figure 2:**
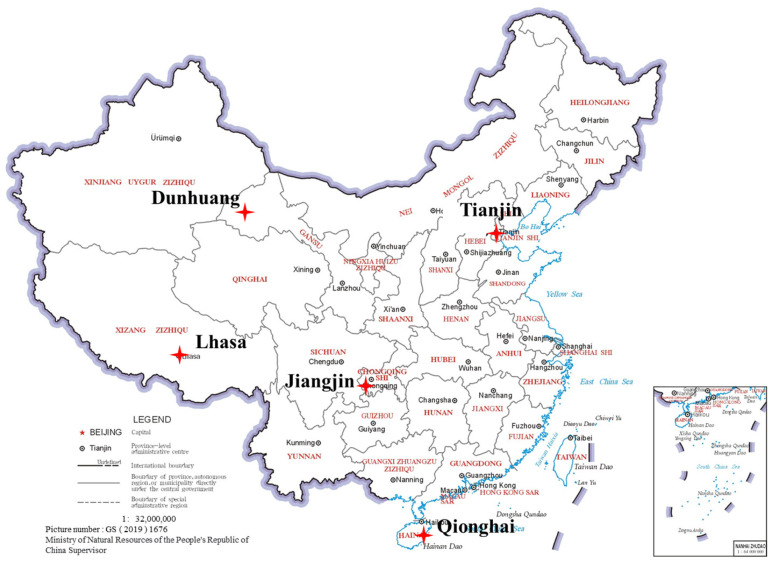
The distribution of the five atmospheric exposure sites.

In this study, atmospheric corrosion monitoring experiments were carried out at five standard exposure sites of the national environmental corrosion platform. The specific locations are shown in Table 2. The distribution of the installation sites for corrosion monitoring sensors and corrosion coupons at the five experimental stations is shown in Figure 2.

The exposure test lasted for 12 months. The corrosion coupons were retrieved in 3 months, 6 months and 12 months, respectively, and the corrosion products of carbon steel were removed in 18 wt.% hydrochloric acid solution with a wire brush according to the standard treatment of ISO8407 C.3.53 [23]. After removing the corrosion products, the average mass loss was calculated by the difference in the weight of the coupons before and after the exposure experiments.

### 2.4. Rust Characterization

Raman spectroscopy (LabRAM HR Evolution, HORIBA Jobin Yvon, Paris, France) was used to determine the composition of the corrosion products, with a laser wavelength of 532 nm, a power of 50 mW, and an exposure time of 10 s. The scanning range was within 0~1800 cm^−1^, with a linear scan range of 200 μm and a sampling dimension of 1 point per 90 μm. The results were analyzed using NanoScope Analysis 2.0 software.

Laser Raman spectrometer (LabRAM HR Evolution, HORIBA Jobin Yvon) was used to analyze the components of the rust layer of carbon steel in different atmospheric environments, with a laser wavelength of 532 nm, a power of 50 mW, and an exposure time of 10 s. The scanning range was within 0~1800 cm^−1^, with a linear scan range of 200 μm.

Macro images of post-test specimens were captured with a digital camera. The cross-section images of the carbon steel rust layer were observed and analyzed using field emission scanning electron microscopy (Gemini SEM 500, ZEISS, Oberkochen, Germany), and EDS was employed to determine the elemental composition of these rust layers.

## 3. Results

### 3.1. Corrosion Sensor Results

**Figure 3:**
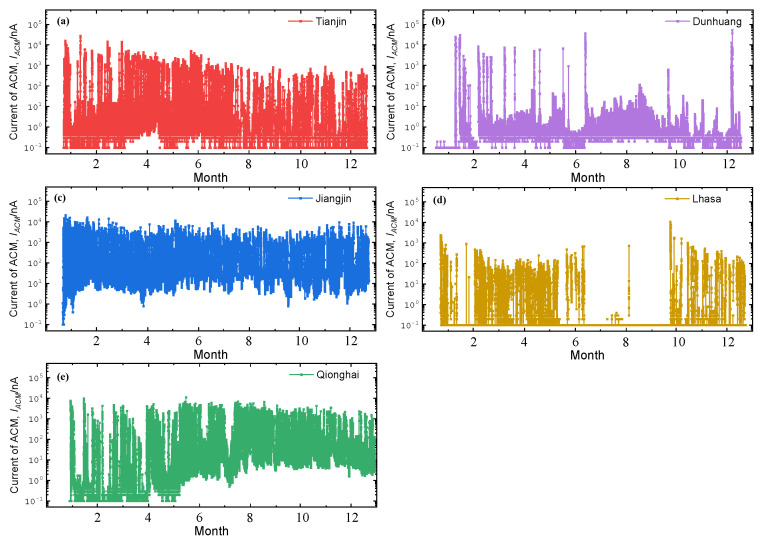
One-year corrosion monitoring current data for five sites: (**a**) Tianjin, (**b**) Dunhuang, (**c**) Jiangjin, (**d**) Lhasa, and (**e**) Qionghai.

Figure 3 shows the output currents from the corrosion sensors (*I_CS_*) at five locations over the entire 1-year test period. The corrosion behavior in each environment exhibits a high degree of volatility. According to Equation (1), the difference between the five atmospheric corrosion sites can be measured by comparing the corrosion monitoring power output (*Q_CS_*) [20] by multiplying the *I_CS_* by 1 min (data acquisition interval) over the test time.

(1) $$Q_{\mathrm{CS}}=\sum I_{\mathrm{CS}}\times 1\;m\mathrm{in}$$

**Figure 4:**
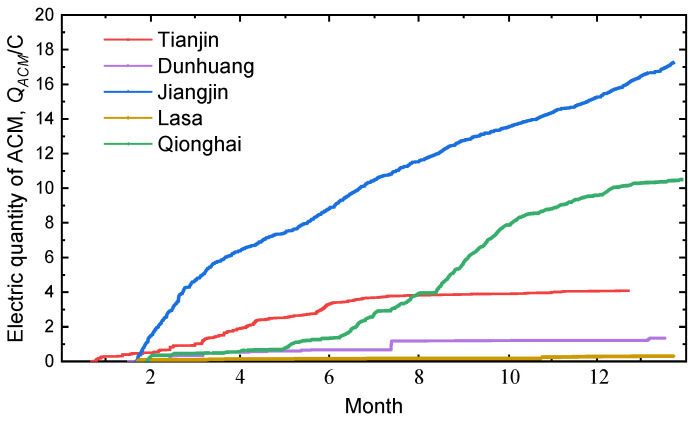
Real-time output intensity of the one-year exposure test corrosion monitoring sensor.

Figure 4 shows the real-time output intensity of corrosion monitoring sensors from five regions during a one-year exposure test. Jiangjin shows rapid increases in electric quantity, indicating significant corrosion, while Lhasa’s electric quantity rises more gradually. Dunhuang remains mostly stable with slight increases at the end, while Lhasa experiences slower corrosion, reflected in its steady curve. Lhasa exhibits minimal variation, indicating minimal corrosion activity. The electric quantity from corrosion sensors in different sites is in the following order: Jiangjin > Qionghai > Tianjin > Dunhuang > Lhasa. The differences highlight varying corrosion conditions across regions.

**Figure 5:**
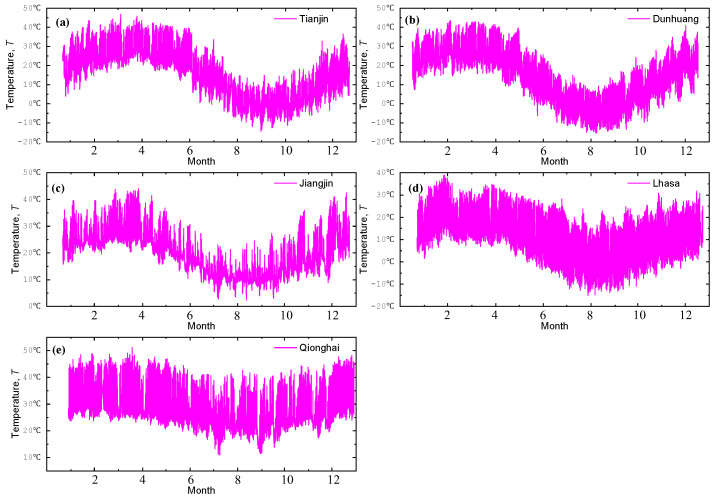
One-year atmospheric temperature data for five sites: (**a**) Tianjin, (**b**) Dunhuang, (**c**) Jiangjin, (**d**) Lhasa, and (**e**) Qionghai.

The real-time temperature monitoring data from the five sites are shown in Figure 5. The temperature trends at the five locations are consistent, with a general pattern of initially decreasing and then increasing. The temperature variation rate at Qionghai is relatively small, remaining consistently above 20 °C. In contrast, Tianjin, Dunhuang, and Lhasa exhibit significant fluctuations, with the difference between the highest and lowest temperatures reaching over 50 °C.

**Figure 6:**
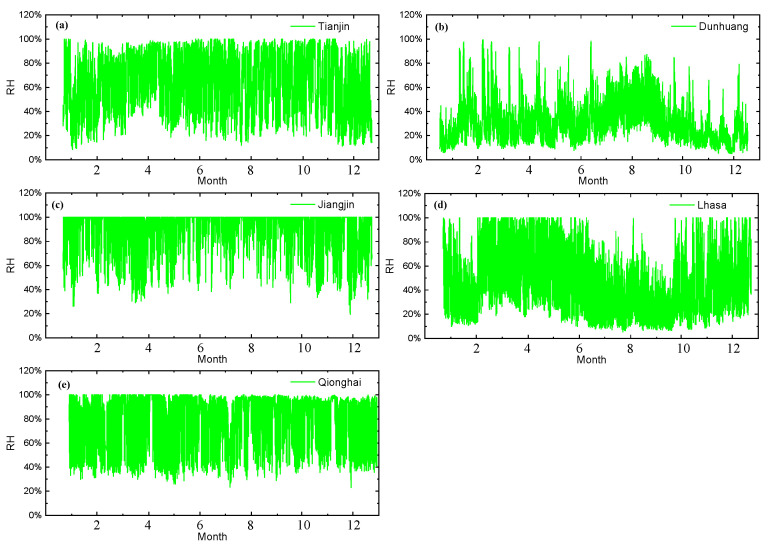
One-year relative humidity data for five sites: (**a**) Tianjin, (**b**) Dunhuang, (**c**) Jiangjin, (**d**) Lhasa, and (**e**) Qionghai.

The real-time relative humidity data are shown in Figure 6. At Tianjin, Jiangjin, and Qionghai, the relative humidity remains between 20% and 100%. Dunhuang exhibits higher average relative humidity from June to October, whereas Lhasa shows the opposite trend during the same period, with humidity levels lower than in other months.

Combined with the real-time monitoring data of temperature and relative humidity from the five locations of the previous study [20], the instability of the outdoor dynamic atmospheric environment within one year can be clearly seen, which in turn leads to the high volatility of the corrosion monitoring current data. The stability characteristics of the indoor solution environment are difficult to simulate the authenticity of the outdoor environment.

### 3.2. Rust Layer Characterization

**Figure 7:**
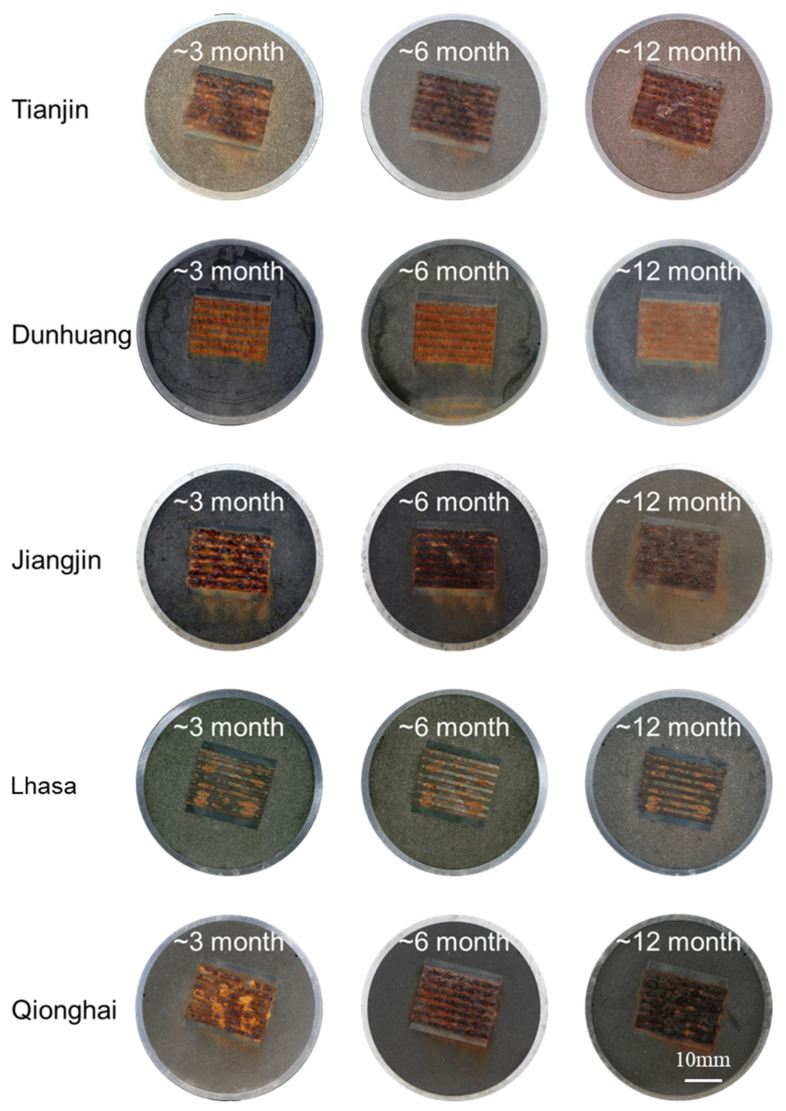
The macroscopic morphology of corrosion sensors at different locations across five sites after one year was studied.

**Figure 8:**
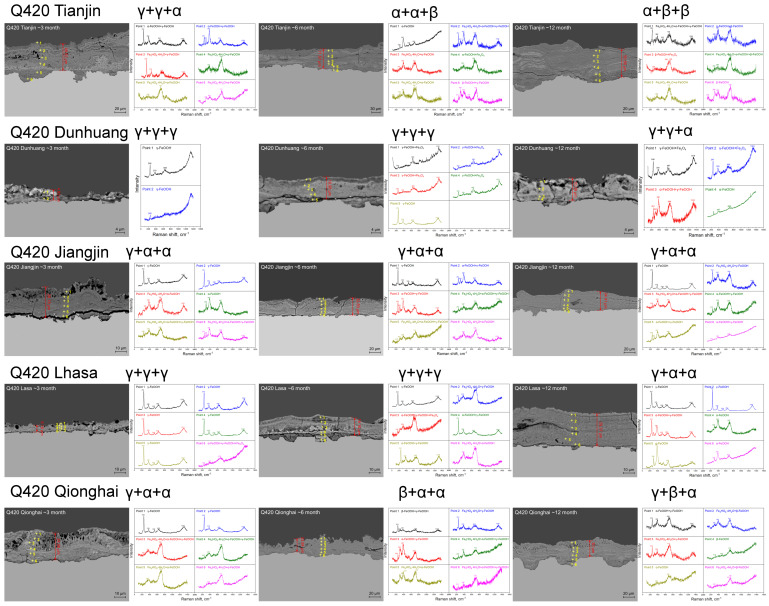
Microscopic Raman diagram of rust layer cross-sections with different exposure cycles at five sites studied in previous research.

**Figure 9:**
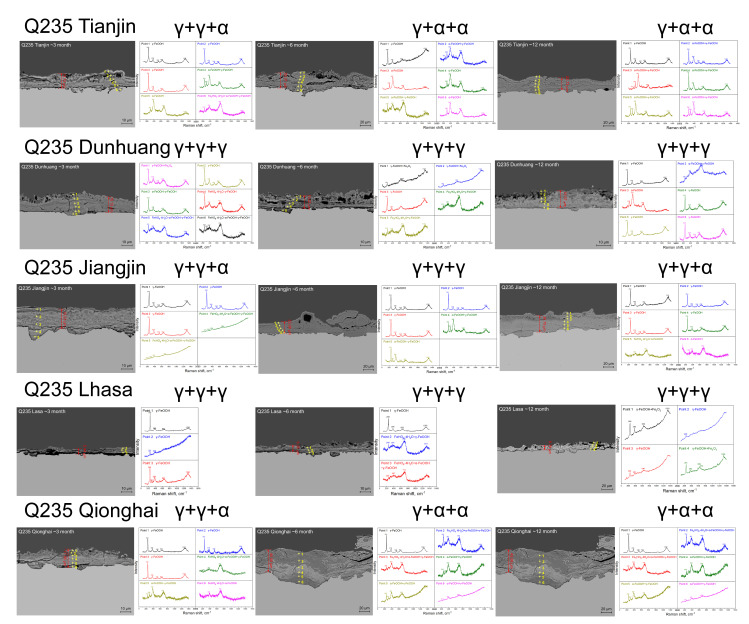
Raman micrograph of rust layer cross-sections with different exposure cycles at five sites in this study.

Figure 7 shows the macroscopic surface topography of corrosion sensors at five sites in this study after different exposure cycles. It can be seen from the figures that the main corrosion form is uniform corrosion, so it is considered that the thickness of the rust layer is uniformly distributed on the sample surface during the corrosion process, which ensures the statistical significance of the rust layer thickness. Figure 8 and Figure 9, respectively, show the rust layer thickness and phase analysis of the rust layer for Q420 weathering steel and carbon steel in different cycles at the five sites. As shown in Figure 8, the rust layer phase structures of Q420 steel vary under different humidity conditions. In regions with higher average humidity, such as Jiangjin and Qionghai, α-FeOOH forms earlier, while only γ-FeOOH is generated in regions with lower average humidity, like Dunhuang and Lhasa. Furthermore, as illustrated in Figure 9, Q235 steel exhibits the same pattern of phase evolution across all five regions during service.

At the same time, Raman spectroscopy was used to measure six points uniformly from the outside to the inside of the rust layer for each period of the sample (samples with a rust layer less than 10 μm can appropriately reduce the number of points, such as Dunhuang, Lhasa); the reference displacement is controlled within 1600 cm^−1^ at each point, and the spectral acquisition time is 60 s. The phase calibration is carried out according to the position of the Raman peak of the corrosion products listed in Table 3 [24], where the value marked in black is the main peak.

Raman measurements of different exposure cycles at six sites [20], and Raman measurements at different exposure cycles at the five sites in this study are shown in Figure 8 and Figure 9.

**Figure 10:**
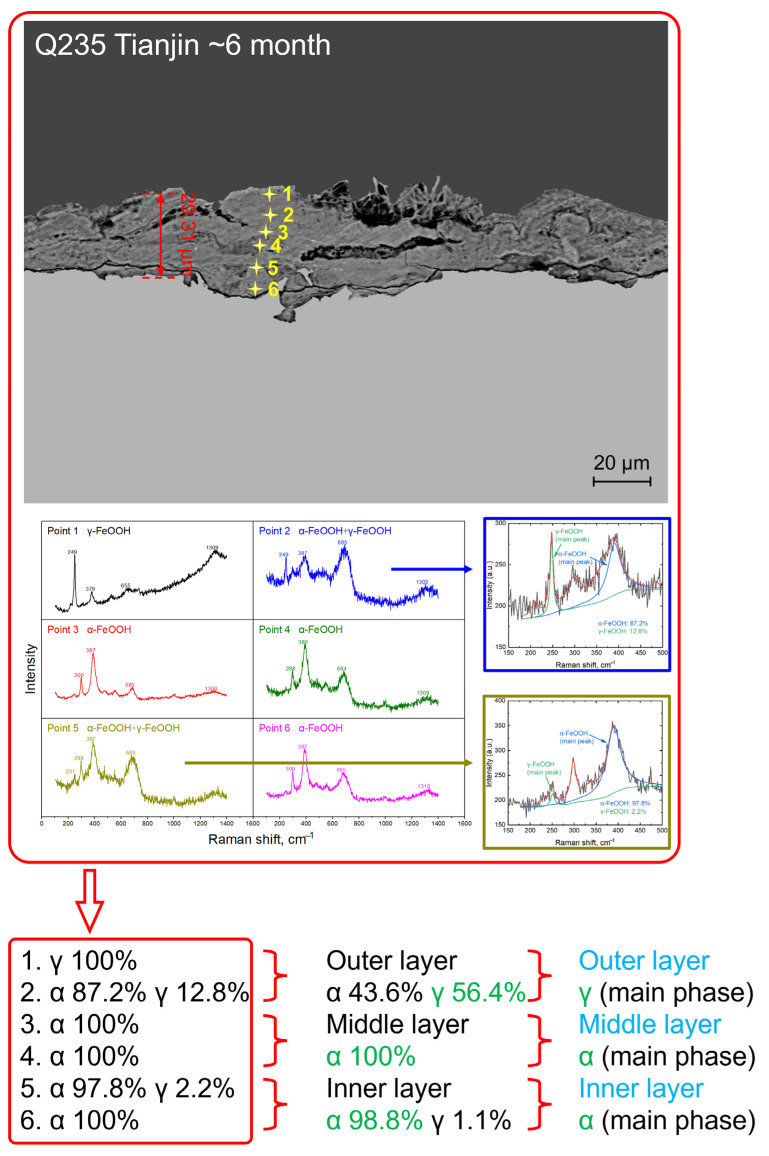
Determination process of the rust layer structure in Tianjin for 6 months.

After calibrating the Raman spectra, the main phases of the rust layer in the outer, middle, and inner layers were determined through semi-quantitative analysis according to the fitting of the main peak intensity [25]. The process of determining the phases of the rust layer is shown in Figure 10. According to the basic Raman data after the calibration of the substance, the area fitting of the main peak strength (α-FeOOH is 387 cm^−1^, γ-FeOOH is 251 cm^−1^) at the test point containing multiple phases, and the different substance content percentages at each test point were calculated to be 87.2% for the α-FeOOH content at the second point and 97.8% for the α-FeOOH content at the fifth point. The two points measured on the outside, middle, and inside layers were accumulated and averaged, and the proportion of contained substances (α-FeOOH and γ-FeOOH) was calculated respectively (the content of γ-FeOOH in the outer layer was 56.4%, and the content of α-FeOOH in the inner layer was 98.9%). The phase with the largest content in the calculation results is considered to be the main rust layer component of the rust layer (the outer layer is γ-FeOOH, the middle layer is α-FeOOH, and the inner layer is α-FeOOH).

### 3.3. Corrosion Resistance Analysis

**Figure 11:**
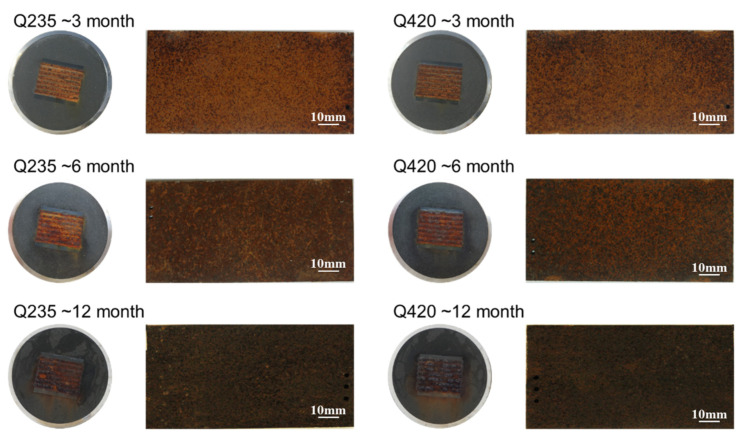
Macromorphology of corrosion sensors and coupons of Q235 carbon steel and Q420 weathering steel for different exposure cycles in Tianjin.

After exploring the influence of the rust layer on atmospheric corrosion through corrosion monitoring technology, Q235 carbon steel and Q420 weathering steel were released in Tianjin to evaluate their corrosion resistance properties. After one year of exposure experiments, the surface morphologies of Q235 carbon steel and Q420 weathering steel and corrosion sensors subjected to corrosion at the Tianjin seaside for 3 months, 6 months, and 12 months are shown in Figure 11. It can be seen from the figure that the color of the rust layer on the corrosion sensor and the corrosion coupon is consistent across each part, and a uniform corrosion morphology can be observed. After 12 months of corrosion, the rust layer on the Q420 corrosion sensor is slightly thicker than that on the Q235 corrosion sensor.

**Figure 12:**
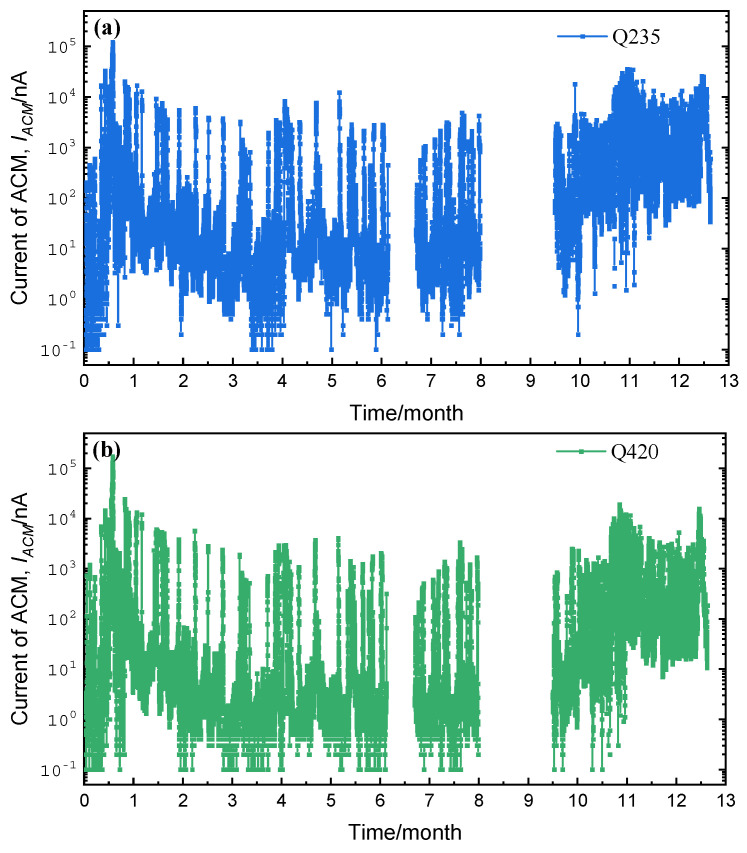
One-year corrosion monitoring current data in Tianjin: (**a**) Q235 carbon steel and (**b**) Q420 weathering steel.

The real-time output currents of the corrosion sensors of Q235 carbon steel and Q420 weathering steel at the Tianjin seaside are shown in Figure 12.

The collected corrosion monitoring data are integrated using Equation (2) to obtain the output power of the corrosion sensor, and then converted using Equation (2) to obtain the real-time corrosion mass loss of the two metal materials [16]:

(2) $$m=36.19\times {Q_{ACM}}^{0.73}$$

In the equation, *m*—weight loss of corrosion coupons, g m^−2^, and *Q_ACM_*—Corrosion sensor output charge, C.

**Figure 13:**
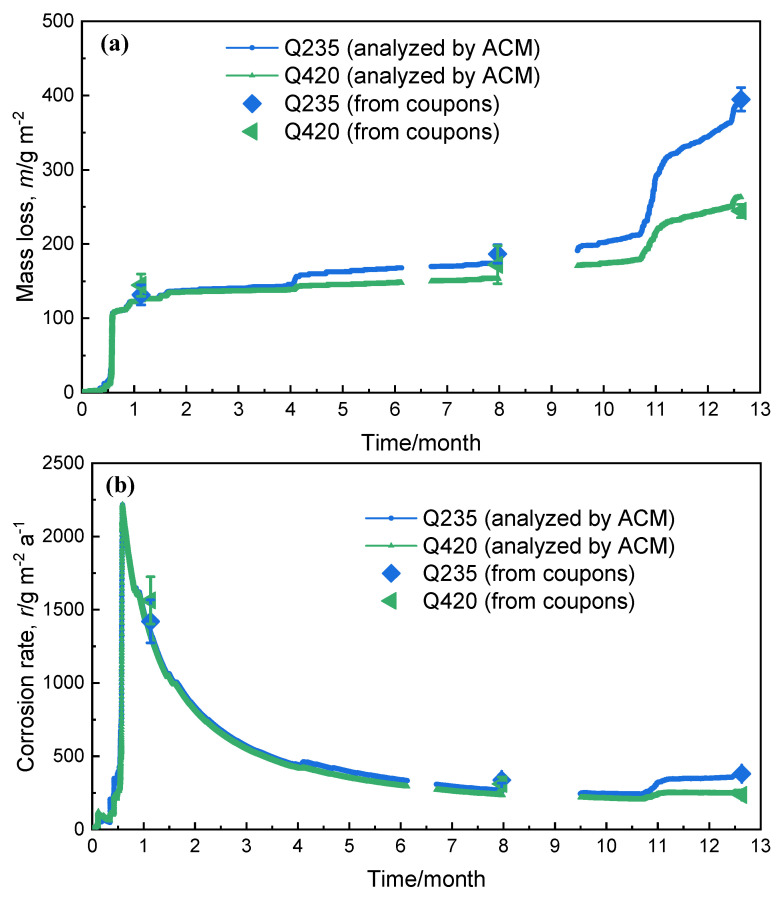
Corrosion monitoring data of Q235 carbon steel and Q420 weathering steel: (**a**) real-time mass loss of corrosion coupons, and (**b**) real-time corrosion rate.

The calculation results are shown in Figure 13. From Figure 13a, it is observed that the corrosion rate of Q420 weathering steel is basically the same as that of Q420 carbon steel at 3 months, indicating that its corrosion resistance is not reflected at all, and the corrosion rate at 6 months is slightly lower than that of Q235 carbon steel, but it is not obvious. When corrosion progresses to 12 months, the corrosion resistance of Q420 weathering steel is fully reflected by the trend of increasing corrosion volume. In addition, they all reflect the rule that the corrosion rate rises first and then decreases in the outdoor atmospheric environment. Figure 13b shows the real-time corrosion rates of the two metal materials. It can be seen from Figure 13 that the weight loss data after analysis of the corrosion monitoring data is very close to that of the corrosion coupons. It is speculated that the reason may be that the carbon content of Q235 and Q420 steel is not very high, so the anodic carriers for corrosion are similar and mainly ferrite. As a results, the empirical equation of corrosion monitoring output power converted into weight loss on corrosion coupons is very close. This phenomenon also verifies the adaptability of Equation (2) in the field of low alloy steel, and proves that corrosion monitoring technology has the potential to replace the traditional corrosion coupons in atmospheric corrosion research of low alloy steel.

**Figure 14:**
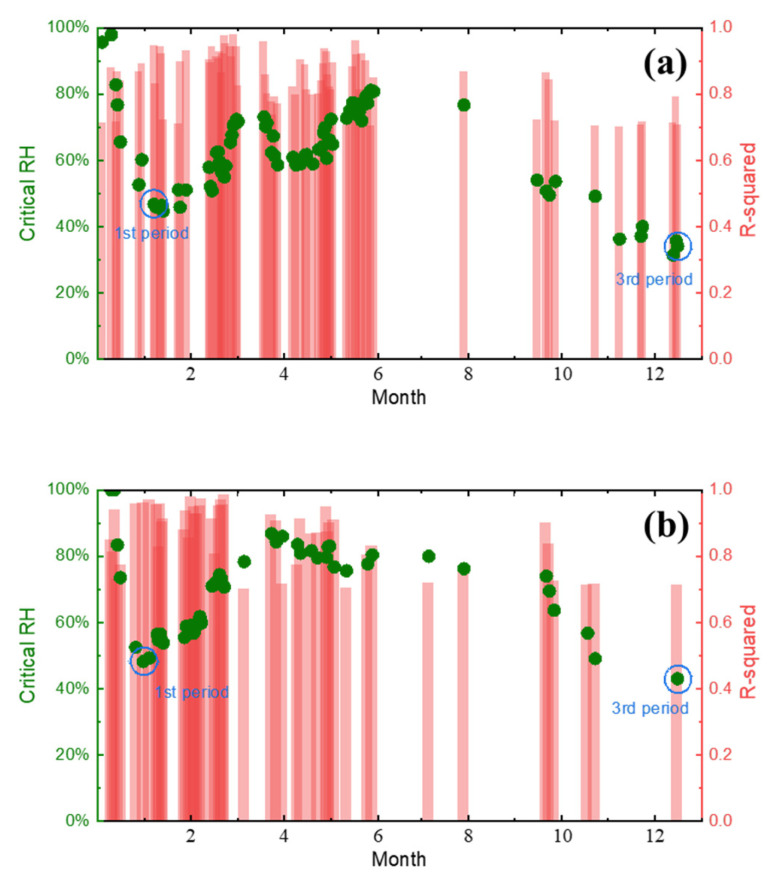
Daily corrosion-critical humidity values of different metal materials in Tianjin: (**a**) Q235 carbon steel; (**b**) Q420 weathering steel.

**Figure 15:**
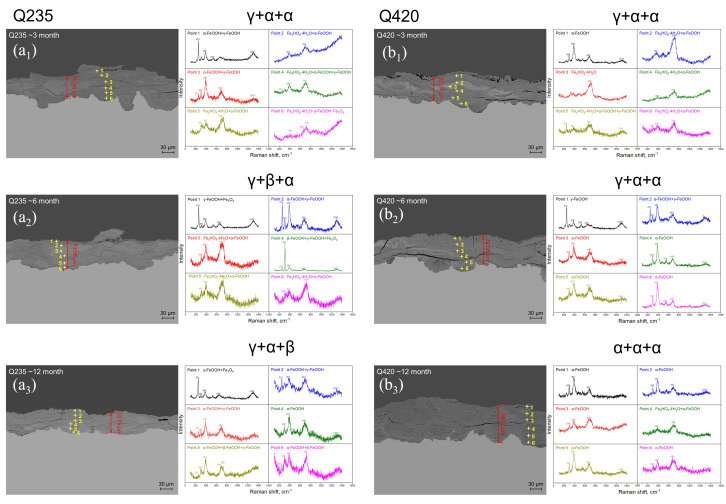
Microscopic Raman diagram of the rust layer cross-section of Q235 carbon steel and Q420 weathering steel in different exposure cycles in Tianjin: (**a_1_**) Q235 carbon steel for 3 month; (**a_2_**) Q235 carbon steel for 6 month; (**a_3_**) Q235 carbon steel for 12 months; (**b_1_**) Q420 weathering steel for 3 month; (**b_2_**) Q420 weathering steel for 6 month; (**b_3_**) Q420 weathering steel for 9 month.

The daily critical humidity values of the two metal materials at the Tianjin test station are shown in Figure 14. The corrosion coupons were recovered at 3 months, 6 months, and 12 months, respectively, to determine the thickness of the rust layer and the type of rust layer structure of the sample, as shown in Figure 15. It can be clearly observed from Figure 15(a_1_,b_1_) that when the corrosion progresses to 3 months, the rust structure type of Q235 carbon steel and Q420 weathering steel is the same, both γ + α + α phases. However, when the corrosion progresses to 6 months, the rust layer structure of the two materials began to change. Figure 15(a_2_) shows that the rust layer structure with the β-FeOOH phase as the main phase appears in the Q235 carbon steel rust layer, while Figure 15(b_2_) shows that the rust layer structure in Q420 weathering steel remains unchanged. This may also be the reason why Figure 15 shows that the trend of mass loss between the two materials at month 6 is inconsistent. When the corrosion progresses to 12 months, Figure 15(a_3_) shows that the Q235 carbon steel rust layer still retains β-FeOOH, but the rust structure of Q420 weathering steel evolves from γ + α + α to the α + α + α structure in Figure 15(b_3_). It has been analyzed that α-FeOOH not only has a good barrier effect on the solution environment, but also has lower porosity and higher density, which is also conducive to resisting the corrosion of the thin liquid film formed by temperature and humidity changes. Therefore, compared with the γ + α + α structure, the rust layer structure of α + α + α is further improved in resistance to atmospheric corrosion in non-rainfall conditions.

Cl^-^ needs to accumulate in the rust layer to promote corrosion, especially during long-term testing [18,26]. Therefore, the presence of β-FeOOH in Q235 carbon steel was not observed in Figure 15(a_1_), but β-FeOOH was significantly detected at 6 months. Unlike Q235 carbon steel, Q420 weathering steel contains 0.4% Ni and 0.36% Cu. Ni atoms can participate in the initial corrosion process and exist in the form of a small amount of Ni(OH)_2_ and NiO in the rust layer; in the dry–wet cycle of day–night alternation or rainfall evaporation, the dispersed Ni(OH)_2_ and NiO in the rust layer can react with Fe(OH)_2_ to form NiFe_2_O_4_ of the spinel phase in the rust layer [24]. On the one hand, fine NiFe_2_O_4_ particles provide many points for the nucleation of α-FeOOH and γ-FeOOH, further promoting the transition from γ-FeOOH to nanoscale α-FeOOH [27]. Therefore, the rust layer in Q420 had more α-FeOOH compared with that of the Q235 steel after 12 months, as shown in Figure 15(b_3_). On the other hand, the grain boundary stability of the rust layer containing NiFe_2_O_4_ is better than that of the rust layer containing only FeOOH [28,29], which in turn effectively resists the invasion of Cl^−^ and prevents its enrichment inside the rust layer. It is inferred from the Raman measurement results that the mechanism of the Ni element promoting the transition from γ-FeOOH to α-FeOOH has promoted the evolution of Q420 weathering steel from γ + α + α in Figure 15(b_1_) to the α + α + α rust layer structure in Figure 15(b_3_). Studies have also shown that when the mass fraction of Ni in steel is 3%, because Ni is enriched in the inner rust layer, the rust layer formed under marine and atmospheric conditions has cation selectivity, which inhibits the penetration of anions through the rust layer and the steel matrix foundation, resulting in better corrosion resistance of the steel compared with that of traditional weathering steel [29]. However, the content of Ni in this study is much lower than 3%, so it is speculated that this mechanism is not the main corrosion resistance mechanism of this steel. The 0.36% Cu element in this weathering steel can not only activate the cathode region on the surface of the steel matrix, passivate the anode region on the steel surface, protect the steel matrix, improve the corrosion resistance of the steel, and also help the formation of an inner rust layer with good continuity and compactness [30]. The dual mechanism of these two elements makes the rust layer denser and resists the infiltration of chloride ions.

**Figure 16:**
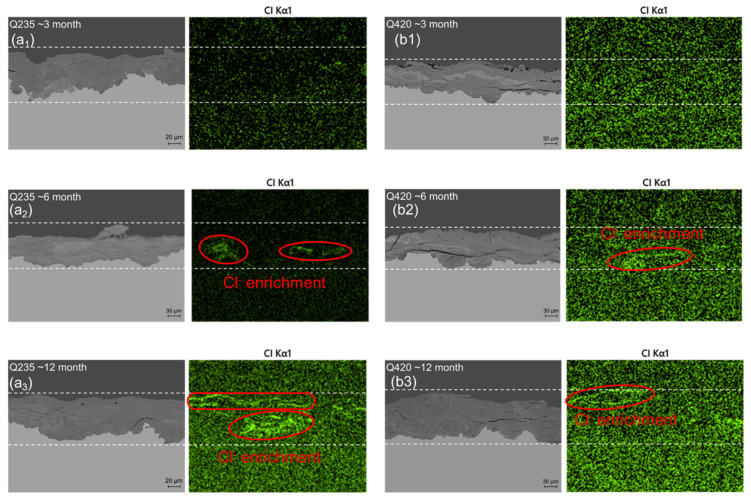
Chloride energy spectrum of Q235 carbon steel and Q420 weathering steel coupons with different corrosion cycles. (**a_1_**) Q235 carbon steel for 3 month; (**a_2_**) Q235 carbon steel for 6 month; (**a_3_**) Q235 carbon steel for 12 months; (**b_1_**) Q420 weathering steel for 3 month; (**b_2_**) Q420 weathering steel for 6 month; (**b_3_**) Q420 weathering steel for 9 month.

Figure 16 shows the chloride energy spectrum of the cross-sections of the two materials with different corrosion cycles in Tianjin. It can be clearly seen that at 3 months, Cl^−^ enrichment did not occur in the rust layer of the two materials, and because the influence of Cl^−^ on the structure of the rust layer had not yet appeared, the rust layer structure in Figure 16(a_1_,b_1_) was consistent. After 6 months of corrosion, the rust layer of both materials showed little Cl^-^ enrichment. However, when the corrosion progressed to 12 months, the Cl^-^ enrichment phenomenon in Q235 carbon steel was very obvious, penetrating into the bottom layer, which also led to the detection of the β-FeOOH phase in the inner layer of the rust layer in Figure 16(a_3_). Ma et al. [18] and Alcántara et al. [31] also observed the lag of chloride ions in outdoor environments in promoting the occurrence of corrosion processes. Correspondingly, the rust layer of Q420 weathering steel performed well when the corrosion progressed to 12 months. The rust layer of the α + α + α structure shielded the Cl^−^ enrichment phenomenon on the surface layer well, effectively protected the steel substrate, and proved the superiority of the corrosion resistance of the weathering steel.

## 4. Discussion

### 4.1. Critical Humidity Analysis of the Test Sites

The cathodic reduction in iron and steel materials reduces the corrosion product (FeOOH), which contains Fe^3+^, to the conductive ferric oxide. The reaction is as follows:

(3) $$Fe\to {Fe}^{2+}+{2e}^{-}$$

(4) $$3\gamma FeOOH+H^{+}+e^{-}\to {Fe}_{3}O_{4}+2H_{2}O$$

In addition, the bivalent iron ions in the rust layer can also react with y-FeOOH to form Fe_3_O_4_.

(5) $${Fe}^{2+}+2\gamma FeOOH={Fe}_{3}O_{4}+2H^{+}$$

(6) $$4{Fe}_{3}O_{4}+O_{2}+2H_{2}O=9\gamma FeOOH$$

Therefore, the corrosion process of metal is

(7) $$\;\;\;\;\;\;\;4Fe\left(s\right)+{4(FeOOH)}_{rust}+2H_{2}O+3O_{2}=4FeOOH+4{\left(FeOOH\right)}_{rust}$$

**Figure 17:**
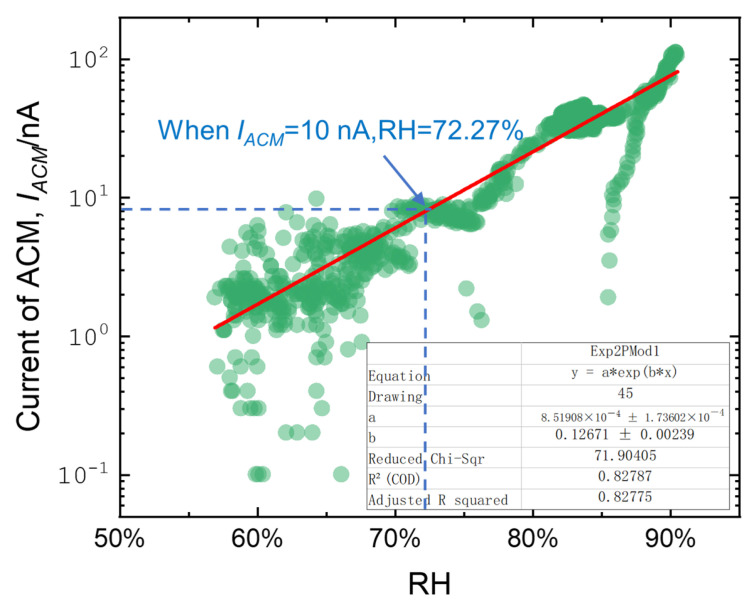
The fitting curve of RH and corrosion monitoring current for one day at Qionghai Station.

The method of obtaining the critical humidity for corrosion takes the data of Qionghai atmospheric station as an example:

In the monitoring data from 12 o’clock on 21 October to 12 o’clock on 22 October, data with temperature > 0 °C and 0% < RH < 90% were selected to reduce the impact of rainfall on the corrosion sensor, and the corrosion monitoring data of the subsequent fitting period were kept in a non-rainfall state as much as possible. As shown in Figure 17, refer to the fitting Equation (8) for the delimited range of data:

(8) $$I_{cs}=A\times {exp}^{B\times RH}$$

In the equation, *I_CS_*—corrosion sensor real-time output current, nA; *RH*—relative humidity of the atmosphere, %; and A, B—fitted constants.

Through Equation (8), the fitting equation of *I*_CS_ and RH on the day was obtained, and the fitting degree R^2^ on the day of fitting was 0.83. It was confirmed that the data noise during the fitting process was acceptable, and the fitting equation could be used for further derivation.

According to the fitting equation, the RH value (72.27%) corresponding to *I_CS_* at a certain value (10 nA) is reversed, which represents the critical humidity of atmospheric corrosion of carbon steel on that day. After several fitting attempts at the five new sites in this study, it was found that when the *I_CS_* value was taken at 50 nA, the sensor was still in the early stage of corrosion because the atmospheric environment in Lhasa and other locations was too weak, and the critical humidity of corrosion was close to 100% for extended periods, making it difficult to show any changes. When the *I_CS_* value was taken as 1 nA, due to the strong corrosive atmospheric environment in Tianjin and other locations, the critical humidity of corrosion was close to 0% for a long time during some periods. Therefore, in this study, the relative humidity corresponding to the *I_CS_* value of 10 nA is used as the critical humidity for corrosion.

**Figure 18:**
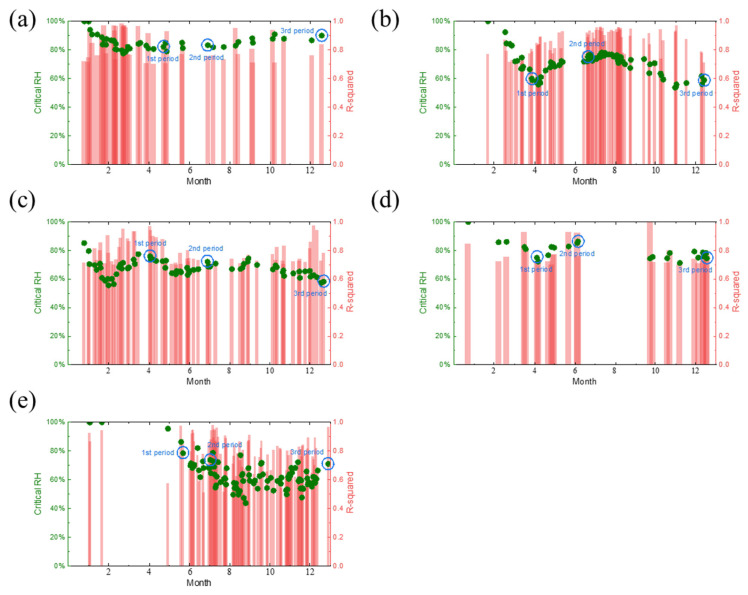
Daily critical humidity values for corrosion at five sites in this study: (**a**) Tianjin, (**b**) Dunhuang, (**c**) Jiangjin, (**d**) Lhasa, and (**e**) Qionghai.

Combined with the daily corrosion monitoring current data and RH data, the critical humidity of corrosion in Tianjin, Dunhuang, Jiangjin, Lhasa, and Qionghai is shown in Figure 18 based on fitting derivation.

Due to the complexity of climatic conditions, it is impossible to guarantee that the data within the selectable range on a given day are reliable; therefore, the derived values with a fitting degree R^2^ less than 0.7 were excluded. It can be clearly seen from the figure that at the beginning of monitoring, the critical humidity of corrosion at all locations was close to 100%, indicating that the smooth electrode surface had difficulty absorbing water from the air, and that the accumulation of the rust layer during the early stage of corrosion played a significant role in promoting the atmospheric corrosion of carbon steel. In addition to the sites of Qionghai, the four sites in Tianjin, Dunhuang, Jiangjin, and Lhasa showed a sharp decrease in the critical humidity at the initial stage of exposure, followed by a slight recovery trend. It is speculated that this phenomenon is due to the alternation of wind and rain, and of cold and heat in the outdoor environment, which subsequently peels off the unstable, loose rust layer formed on the surface of the corrosion sensor during the early stage of corrosion. These effects reduce the porosity of the surface of the rust layer, weaken the water absorption of the rust layer, and finally lead to the rise of the critical humidity of corrosion.

### 4.2. Phase Structure Analysis of the Rust Layer

**Figure 19:**
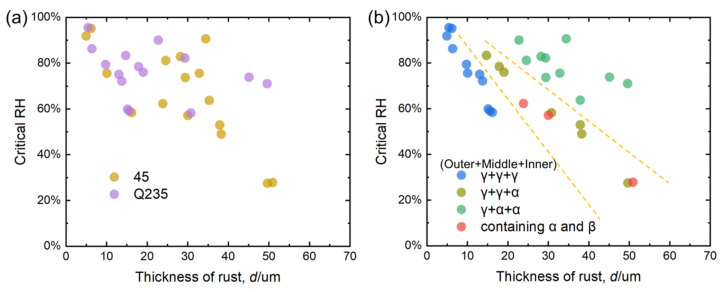
Relationship between the thickness of the carbon steel rust layer and the critical humidity of corrosion: (**a**) division of different steel grades; (**b**) division of different rust layer structure types.

Combined with the original Raman measurement results in Figure 11 and Figure 12, the main rust components of the outer, middle, and inner layers of the rust layer in different corrosion cycles at different sites were determined by fitting derivation, and the results were marked on the upper side of the Raman measurement results in Figure 11 and Figure 12 in the order of outer layer + middle layer + inner layer. Finally, the thickness of the rust layer and the critical humidity were divided into different steel grades and different rust layer structure types, and the results are shown in Figure 19. Figure 19a shows that the relationship between the thickness of the rust layer and the critical humidity is roughly the same for Q420 steel and Q235 carbon steel, but the relationship is not obvious. Therefore, it is not easy to judge the difficulty of atmospheric corrosion in non-rainy conditions simply based on the type of carbon steel and the thickness of the rust layer. Figure 19b clearly distinguishes the effect of different types of rust structure on the critical humidity of atmospheric corrosion.

Figure 19b shows that when the thickness of the rust layer is less than 70 μm, the critical humidity of corrosion under each rust layer structure decreases with the increase in the thickness of the rust layer, indicating that the rust layer has played a role in promoting atmospheric corrosion in a non-rainfall state. As mentioned earlier, the growth of the rust layer generally plays a role in first promoting and then inhibiting the corrosion reaction [32]. After the initial stage of corrosion, a relatively stable rust layer has been formed, and further corrosion reactions on the metal surface require reactants to penetrate through the rust layer, and the corrosion products also need to be exported outward. With the progress of corrosion, the thickness and density of the rust layer can hinder the transfer efficiency of dissolved oxygen in the thin liquid film during the corrosion reaction, resulting in a decrease in the diffusion of dissolved oxygen from the thin liquid film surface to the metal surface per unit time. The dissolved oxygen concentration in the metal surface area becomes low, which will inevitably affect the cathode oxygen uptake process [33] and finally inhibit the corrosion reaction and reduce the reaction rate. The phenomenon in the figure shows that for any kind of rust layer structure formed in the outdoor dynamic environment, when the phase structure is stable and the thickness of the rust layer does not change greatly within the range of 70 μm, the rust layer may play a certain barrier effect in the atmosphere of rain, but it cannot play an effective barrier role in the moisture in the atmosphere.

**Figure 20:**
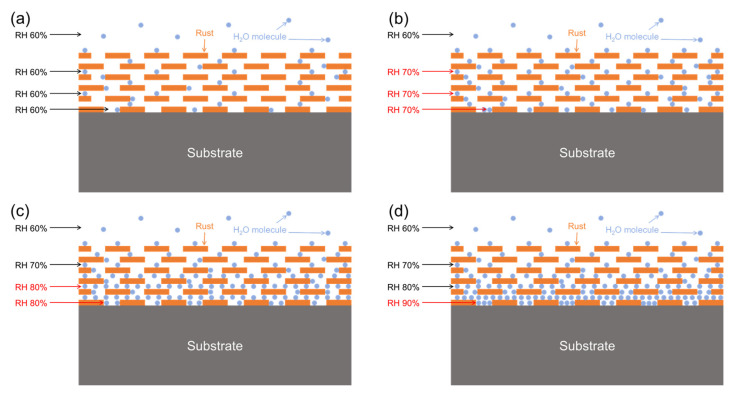
Evolution process model of relative humidity on the substrate surface of carbon steel: (**a**) initial unhygroscopic state, (**b**) initial hygroscopic state, (**c**) infiltration state, and (**d**) final state.

Figure 20 shows the evolution of relative humidity on the surface of carbon steel. The initial state of relative humidity inside the rust layer is shown in Figure 20a. The density of water molecules in the pores of corrosion products inside the rust layer is the same as that of the water molecules in the atmosphere. However, due to the water absorption performance brought by the roughness of the rust layer, the rust layer has a stronger ability to attract water molecules, resulting in the relative humidity of the surface being higher than that of the atmosphere. The hygroscopic state shown in Figure 20b evolves. Because the process of adsorbing water molecules on the surface of the object is dynamic, accompanied by the process of water evaporation, only the timely diffusion of evaporated water molecules can maintain the constant relative humidity of the surface of the object. Compared with the outer rust layer with diffusion space on both sides, the inner rust layer is closer to the metal substrate, and the downward spreading space substrate is blocked, which makes it difficult for evaporating water molecules to escape in time, so that the relative humidity of the surface of the inner layer of the rust layer is further increased, and the formed state is shown in Figure 20c. Figure 20d shows the final state after the whole system reaches dynamic equilibrium. The diffusion space near the rust layer on the metal surface is the most crowded, resulting in the highest relative humidity. The closer the surface of the rust layer is, the closer the relative humidity in the rust layer is to the relative humidity in the atmosphere. In the meantime, Figure 14b shows the fitting results of the data with RH no more than 90% in the accumulated data. It can be seen that the moisture absorption efficiency of the rust layer is very limited, and the relative humidity of 90% of the atmosphere is not enough for the rust layer within 70 μm thickness to form a thin liquid film that allows oxygen diffusion to control the corrosion rate. It is easy for oxygen to diffuse to the surface of the metal substrate through the liquid film, which finally promotes the phenomenon that the critical humidity of corrosion under each rust layer structure decreases with the increase in the thickness of the rust layer.

The data of the γ + γ + γ rust layer structure are distributed on the far left, indicating that the structure has the strongest effect on atmospheric corrosion caused by relative humidity change among the four types of rust layer structures in the statistics, and the data of the γ + α + α rust layer structure are distributed on the far right, indicating that the structure has the weakest effect on atmospheric corrosion caused by relative humidity change. In terms of structure, α-FeOOH is a layered plate structure, and the growth direction of α-FeOOH is oriented in different directions. Not only is the stress generated inside the rust layer small but the sheets are also superimposed and overlaid with each other, so that the formed rust layer has good compactness, continuity, and low porosity [34], which can effectively block the penetration of aggressive ions into the steel matrix and improve the atmospheric corrosion resistance of steel. In contrast, γ-FeOOH is a needle-like form, and the germination is in the form of upward or downward growth. This structure not only increases the internal stress of the entire rust layer, but also increases the spacing between the layers, the cracks and gaps between the rust layers, and the moisture absorption of the rust layer, thereby reducing the critical corrosion humidity of the steel, making the thin liquid film and dissolved oxygen more likely to penetrate into the inner rust layer. It results in a decrease in the atmospheric corrosion resistance of the steel and accelerates the corrosion rate inside the rust layer and the steel matrix. For example, in Figure 8, the cross-sectional morphology of the rust layer in Jiangjin from the 6th to the 12th month shows that the holes and cracks inside are become fewer and fewer, indicating that the internal stress of the rust layer gradually decreases, and the density and stability gradually increase, while the test results show that the structure of the rust layer changes from γ + γ + α to γ + α + α. In terms of electrochemical properties, α-FeOOH is an insulating inactive substance, which is electrochemically and thermodynamically stable, and is also the most stable hydroxyferrooxide (the main component of the protective rust layer) [7]. Relatively speaking, γ-FeOOH has been studied to have very active thermodynamic properties in the rust layer, and it is easy to be reduced by Fe^2+^ to other auxiliary products with relatively stable properties during the wetting process. From the structural water absorption and electrochemical characteristics, it is ultimately determined that the more components of α-FeOOH, the better the protection of the generated corrosion products, and the better the corrosion resistance of steel. Therefore, in the atmospheric environment under non-rainfall states, the γ + γ + γ rust layer structure has the strongest promotion effect on atmospheric corrosion, while the γ + α + α rust layer structure has the weakest promotion effect on atmospheric corrosion.

When the thickness of the rust layer reaches more than 20 μm, it is difficult to evaluate the data of the structure of the γ + γ + γ rust layer, and when the thickness of the rust layer is less than 20 μm, it is difficult to evaluate the data of γ + α + α and the structure of the α and β rust layer, indicating that the γ + γ + γ rust layer structure is more likely to be observed in the early stage of corrosion in the outdoor environment, and when the thickness of rust layer reaches a certain degree, it is easy to evolve to other structures. This phenomenon is mainly caused by the electrochemical properties of different FeOOHs. Wu et al. [24] used Raman spectroscopy to study the reducible properties of corrosion products containing Fe^3+^, and found that the thermodynamically active γ-FeOOH and δ-FeOOH in the rust layer are more likely to be reduced to Fe_3_O_4_ and α-FeOOH by Fe^2+^, while α-FeOOH, which is thermodynamically and kinetically stable, is not easy to be reduced. In addition, the relatively dense sheet structure characteristics of α-FeOOH have a more obvious effect on the physical peeling in the atmospheric environment, while the loose and porous γ-FeOOH has weaker adhesion to the metal matrix. Therefore, with the extension of the corrosion cycle, the amount of α-FeOOH material continues to increase and remains in the rust layer, which promotes the evolution of the rust layer structure.

The structure of the γ + γ + α rust layer is in the same interval as the data distribution containing the α and β rust layer structures, indicating that the β phase rust layer structure is extremely conducive to atmospheric corrosion, and no rust layer structure containing only γ and β as the main phases has been found in the current data. First, the rust layer structure of β-FeOOH is similar to that of γ-FeOOH, showing needle-like and columnar morphology, growing upward or downward, eventually leading to its hygroscopicity similar to γ-FeOOH [31]. The research of Yang et al. [26] shows that the electrochemical activity of β-FeOOH is very strong, and the thermodynamics and kinetics of the rust layer are unstable. So β-FeOOH is easily reduced during the wetting process, resulting in a decrease in the protective performance of the rust layer. Therefore, the rust structure containing β-FeOOH has very limited resistance to atmospheric corrosion, which also leads to the distribution of the rust layer data containing α and β in the middle region of Figure 14b. However, in the current data, no rust layer structure containing only γ and β as the main phases has been found, and it is speculated that the appearance of β-FeOOH is generally caused by the corrosive effect of Cl^−^, and the limited concentration of Cl^−^ in the outdoor atmospheric environment generally promotes the formation of γ-FeOOH in the rust layer at the beginning. After the initial stage of corrosion, the accumulation of a certain concentration of Cl^−^ in the rust layer will promote the formation of β-FeOOH [18,19], and after the initial stage of corrosion, due to the strong adhesion of the rust layer formed by α-FeOOH, it is easier to leave an α-FeOOH structure in the rust layer.

### 4.3. Relationship Between Thickness of Carbon Steel Rust Layer and Critical Humidity of Corrosion

**Figure 21:**
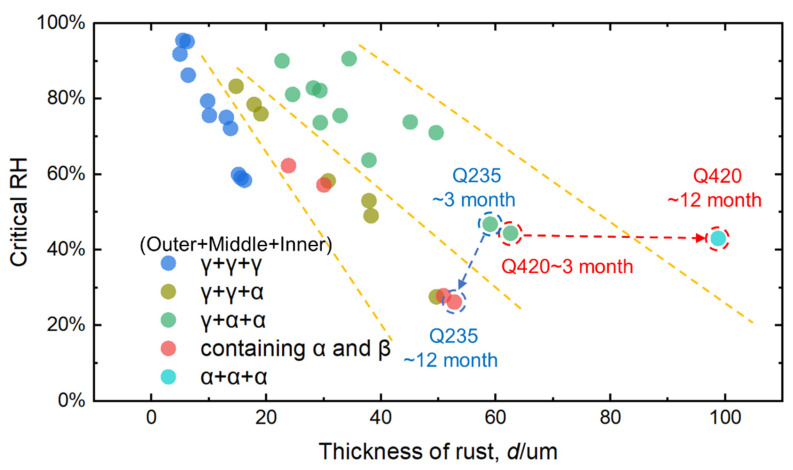
The relationship between the thickness of the carbon steel rust layer and the critical humidity of corrosion is divided into different rust layer structure types.

The rust layer data from Figure 18 and Figure 19 are incorporated into Figure 21. It can be clearly observed that the thickness of the rust layer of Q420 weathering steel reached 100 μm at 12 months, but the corresponding critical humidity did not change significantly, while the thickness of the rust layer of Q235 carbon steel slightly thinned, but the critical humidity decreased significantly, indicating that the key to the corrosion resistance of Q420 weathering steel lies in the transformation of the phase structure of the rust layer, and the difficulty of atmospheric corrosion cannot be evaluated only by the thickness of the rust layer.

At the same time, based on the accumulated rust layer thickness and corresponding corrosion-critical humidity data of ordinary carbon steel and weathering steel accumulated in Figure 21, the empirical formula for the rust layer thickness-corrosion-critical humidity of each rust layer structure was established:

(1) For γ + γ + γ structure:

(9) $${RH}_{Critical}=175.02\times d^{-0.37}$$

where *RH_Critical_* is critical humidity of atmospheric corrosion, %;

*d*—thickness of the rust layer, μm.

The degree of fit R^2^ was 0.87.

(2) For γ + γ + α structure:

(10) $${RH}_{Critical}=674.57\times d^{-0.75}$$

The degree of fit R^2^ was 0.89.

(3) For γ + α + α structure:

(11) $${RH}_{Critical}=448.12\times d^{-0.51}$$

The degree of fit R^2^ was 0.65.

Because there is less data containing α and β structures, these are not fitted.

## 5. Conclusions

In this study, Fe/Cu electrochemical corrosion monitoring technology was used to monitor the atmospheric corrosion of Q235 and Q420 carbon steel at the Tianjin, Dunhuang, Jiangjin, Lhasa, and Qionghai test sites for 12 months. The following conclusions are drawn:

(1) Based on the real-time corrosion monitoring data, a method for calculating the critical humidity of atmospheric corrosion is proposed, and it is found that, in the process of atmospheric corrosion caused by changes in outdoor temperature and humidity, the critical humidity of atmospheric corrosion generally decreases first and then rises. When the thickness of the rust layer is less than 70 μm, the critical humidity of corrosion under each rust layer structure decreases with the increase in thickness, indicating that the rust layer in this thickness range plays a role in promoting atmospheric corrosion in a non-rainfall state.
(2) The rust layer structure with γ-FeOOH as the main phase is easier to observe in the early stages of corrosion, and when the thickness of the rust layer is greater than 20 μm, it tends to evolve into other structures. Compared with α-FeOOH, β-FeOOH has a stronger effect on atmospheric corrosion caused by temperature and humidity changes, and β-FeOOH is not suddenly generated in the outdoor environment but slowly forms in the rust layer over time. The key to improving the corrosion resistance of weathering steel is the transformation of the phase structure of the rust layer, and the difficulty of atmospheric corrosion cannot be evaluated only by the thickness of the rust layer.
(3) The corrosion sensor current and corrosion coupons have the following empirical formula conversion relationship: $m=36.19\times {Q_{ACM}}^{0.73}$. This conversion relationship is applicable to other low-alloy steels, proving that corrosion-monitoring technology has the potential to replace traditional corrosion coupons for atmospheric corrosion research on low-alloy steels.

## Figures and Tables

**Table 1:** Chemical compositions of experimental materials (wt.%).

Steels	C	Si	Mn	S	P	Ni	Cr	Cu	Fe
Q235	0.16	0.078	0.3	0.016	0.014	≤0.01	≤0.01	≤0.01	Bal.
Q420	0.057	0.23	1.12	0.0075	0.009	0.4	0.038	0.36	Bal.

**Table 2:** Coordinates and climate types of five field experiment sites.

Location	Latitude and Longitude	Climate Types	Environmental Corrosive Classification (ISO9223)
Tianjin	117.40° E, 39.55° N	Rural atmosphere	C2–C3
Dunhuang	94.55° E, 40.08° N	Desert hot and dry atmosphere	C1–C2
Jiangjin	106.25° E, 29.32° N	Subtropical humid suburban acid rain environment	C3–C4
Lhasa	91.15° E, 29.48° N	Highland atmospheric environment	C1–C2
Qionghai	110.48° E, 19.24° N	Tropical humid rural environment	C3–C4

**Table 3:** Raman spectral peaks of the phases present in the corrosion products.

Phase	Peak/cm^−1^
γ-FeOOH	166, 217, 251, 310, 350, 378, 529, 655, 713, 1300
α-FeOOH	203, 244, 300, 387, 399, 415, 480, 552, 684,1002, 1113, 1304
β-FeOOH	139, 308, 331, 389, 420, 499, 539, 609, 720, 1410
Fe_3_O_4_	306, 538, 666
Fe_2_O_3_	228, 250, 294, 414, 502, 625, 670, 1330
γ-Fe_2_O_3_	Peak width 339~386, 461~512, 671~717, 1430
Fe_5_HO_8_·4H_2_O	Peak width 700~710

## Data Availability

The raw data supporting the conclusions of this article will be made available by the authors on request.
